# (*S*)-Methyl 2-{(*S*)-2-[bis­(4-meth­oxy­phen­yl)methyl­idene­amino]-3-hy­droxy­propanamido}-3-methyl­butano­ate

**DOI:** 10.1107/S1600536810049032

**Published:** 2010-12-04

**Authors:** Charles M. Keyari, Robin Polt, Gary S. Nichol

**Affiliations:** a Department of Chemistry & Biochemistry, The University of Arizona, 1306 E University Boulevard, Tucson, AZ 85721, USA

## Abstract

The title compound, C_24_H_30_N_2_O_6_, a Schiff base, adopts an extended conformation in which the meth­oxy groups are essentially coplanar with the aromatic ring to which they are bonded (mean planes fitted through the non-H atoms of each methoxyphenyl group have r.m.s. deviations of 0.078 and 0.044 Å) and the angle between mean planes fitted through the aromatic rings is 87.57 (10)°. An intra­molecular N—H⋯N hydrogen bond keeps the imine and amide groups essentially coplanar. A mean plane fitted through these groups has an r.m.s. deviation of 0.0545 Å. Additional O—H⋯O hydrogen bonding parallel with the *a* axis links the mol­ecules into a hydrogen-bonded chain in the crystal. C—H⋯O and C—H⋯π inter­actions are found within the crystal packing. The compound has been assigned the *S*,*S* configuration on the basis of the chemical synthesis, which used pure homotopic l-amino acids, and we have no reason to believe that the compound has epimerized.

## Related literature

For background to our inter­est in developing new synthetic methods towards the synthesis of glycopeptide analogues and related compounds, see: Dhanasekaran *et al.* (2005 ▶); Dhanasekaran & Polt (2005 ▶); Egleton *et al.* (2005 ▶); Lowery *et al.* (2007 ▶); Polt *et al.* (2005 ▶); Keyari & Polt (2010 ▶). For a related structure, see: Wijayaratne *et al.* (1993 ▶).
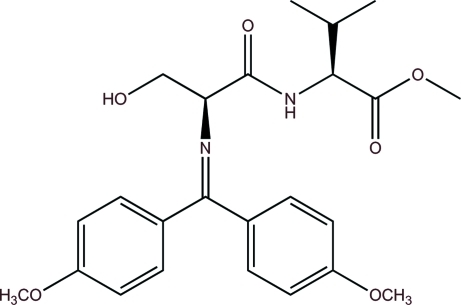


## Experimental

### 

#### Crystal data

C_24_H_30_N_2_O_6_
*M* *_r_* = 442.50
Triclinic, 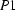

*a* = 5.847 (5) Å
*b* = 8.981 (7) Å
*c* = 11.630 (9) Å
α = 80.456 (11)°
β = 83.922 (11)°
γ = 76.971 (12)°
*V* = 585.2 (8) Å^3^
*Z* = 1
Mo *K*α radiation
μ = 0.09 mm^−1^
*T* = 150 K
0.60 × 0.20 × 0.10 mm

#### Data collection

Bruker SMART 1000 CCD diffractometer
Absorption correction: multi-scan (*SADABS*; Sheldrick, 1996 ▶) *T*_min_ = 0.948, *T*_max_ = 0.991
3801 measured reflections
1965 independent reflections
1484 reflections with *I* > 2σ(*I*)
*R*_int_ = 0.029

#### Refinement

*R*[*F* ^2^ > 2σ(*F* ^2^)] = 0.037
*wR*(*F* ^2^) = 0.107
*S* = 1.09
1965 reflections
301 parameters
5 restraints
H atoms treated by a mixture of independent and constrained refinement
Δρ_max_ = 0.18 e Å^−3^
Δρ_min_ = −0.19 e Å^−3^

### 

Data collection: *SMART* (Bruker, 2007 ▶); cell refinement: *SAINT* (Bruker, 2007 ▶); data reduction: *SAINT*; program(s) used to solve structure: *SHELXTL* (Sheldrick, 2008 ▶); program(s) used to refine structure: *SHELXTL*; molecular graphics: *ORTEP-3 for Windows* (Farrugia, 1997 ▶) and *Mercury* (Macrae *et al.*, 2008 ▶); software used to prepare material for publication: *SHELXTL*, *publCIF* (Westrip, 2010 ▶) and local programs.

## Supplementary Material

Crystal structure: contains datablocks I, global. DOI: 10.1107/S1600536810049032/bh2325sup1.cif

Structure factors: contains datablocks I. DOI: 10.1107/S1600536810049032/bh2325Isup2.hkl

Additional supplementary materials:  crystallographic information; 3D view; checkCIF report

## Figures and Tables

**Table 1:** Hydrogen-bond geometry (Å, °) *Cg*1 is the centroid of the C18–C23 ring.

*D*—H⋯*A*	*D*—H	H⋯*A*	*D*⋯*A*	*D*—H⋯*A*
O1—H1*O*⋯O2^i^	0.84 (1)	1.87 (2)	2.705 (5)	170 (6)
N1—H1*N*⋯N2	0.84 (1)	2.21 (4)	2.641 (5)	112 (4)
C6—H6*B*⋯O1^ii^	0.98	2.49	3.353 (6)	146
C17—H17*C*⋯O3^iii^	0.98	2.53	3.410 (6)	149
C20—H20⋯O3^i^	0.95	2.46	3.222 (6)	137
C16—H16⋯*Cg*1^iv^	0.95	2.52	3.460 (6)	169

Symmetry codes: (i) 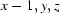; (ii) 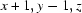; (iii) 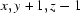; (iv) 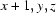.

**Fig. 1.**
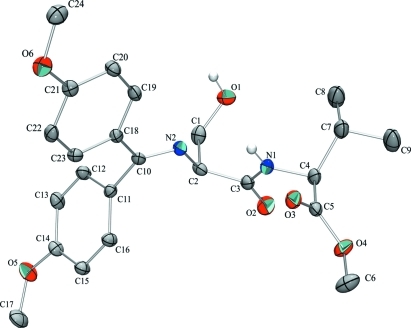
The structure of (I) with displacement ellipsoids at the 30% probability level. C-bound H atoms are omitted.

**Fig. 2.**
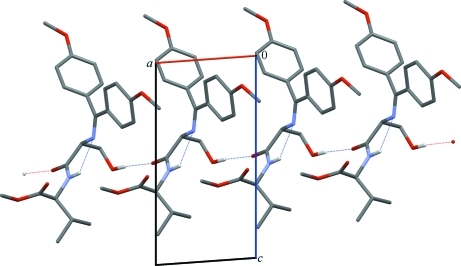
N—H···N and O—H···O hydrogen bonding, indicated by blue dotted lines, in the structure of (I).

## supplementary crystallographic information

### Comment

We have been interested for some time in developing new synthetic methods towards the synthesis of glycopeptide analogues and related compounds (Dhanasekaran *et al.*, 2005; Dhanasekaran & Polt, 2005; Egleton *et al.*, 2005; Lowery *et al.*, 2007; Polt *et al.*, 2005). The crystal structure of a dipeptide Schiff base, (I), was determined as part of our work, and is presented here. In solution such Schiff bases are normally present in equilibrium with an oxazolidine tautomer and we have previously reported the structure of a related compound which crystallized as the oxazolidine form (Wijayaratne *et al.*, 1993).

The molecular structure of (I) is shown in Figure 1. The compound adopts an extended conformation and the molecular geometry is largely unexceptional. This conformation is given added stability by an intramolecular N—H···N hydrogen bond. O—H···O hydrogen bonding parallel with the *a* axis, as shown in Figure 2, connects the molecules into a hydrogen bonded chain. Weak C—H···O and C—H···π interactions are found within the crystal packing, although there is no evidence of face-face aromatic stacking.

### Experimental

*L*-Valine methyl ester HCl salt (1.02 g, 6.11 mmol, 2.0 equiv.), benzyl *N*-[9-(fluorenylmethoxycarbonyl)]-*L*-serinate (1.0 g, 3.06 mmol, 1.0 equiv.), *N*-hydroxybenzotriazole (0.94 g, 6.11 mmol, 2.0 equiv.), *O*-benzotriazole-*N*,*N*,*N*',*N*-tetramethyluroniumhexafluorophosphate (2.32 g, 6.11 mmol, 2.0 equiv.) and 5.5 ml diisopropylethylamine (30.6 mmol, 10.0 equiv.) were stirred overnight in 15 cm^3^ of dichloromethane. The reaction mixture was then washed and concentrated and crystallization from ethyl acetate and hexanes provided white crystals. The crystals were then reacted with 20% piperidine in dichloromethane (15 ml) for 1 h. This was then concentrated and 1 N HCl in methanol was added with stirring at room temperature for 15 min. The solvent was stripped off and bis(4-methoxy)-diarylketimine (0.57 g, 2.36 mmol, 1.0 equiv.) was added to the HCl methyl ester salt and dried over P_2_O_5_ overnight *in vacuo*. Dry acetonitrile (10 ml) was added and stirring started at room temperature and reacted for at least 16 hrs. The crystalline product (I) was obtained by recrystallization from ethyl acetate and hexanes. Yield 0.23 g (0.52 mmol, 21% over 3 steps); mp = 120–122°C. FABMS: C_24_H_30_N_2_O_6_, *m/z* [*M* + H]^+^443.2. For a more detailed description of the overall synthetic procedure see Keyari & Polt (2010).

### Refinement

All H atoms were first located in a difference map. O—H and N—H were refined using an *X*—H distance restraint of 0.84 (1) Å. C-bound H atoms have constrained C—H distances of 0.95 Å, 0.98 Å, 0.99Å and 1.00Å for Ar—H, CH_3_, CH_2 _and CH respectively. All H atoms were refined as riding with *U*_iso_(H) = 1.5 *U*_eq_(C) for methyl groups, while all others were refined with *U*_iso_(H) = 1.2 *U*_eq_(*X*). 1636 Friedel pairs were measured, but due to a lack of significant anomalous dispersion they were merged during final refinement cycles. The compound has been assigned the *S*,*S* configuration on the basis of the chemical synthesis.

### Crystal data

**Table:**

C_24_H_30_N_2_O_6_	*Z* = 1
*M**_r_* = 442.50	*F*(000) = 236
Triclinic, *P*1	*D*_x_ = 1.256 Mg m^−^^3^
Hall symbol: P 1	Melting point: 393 K
*a* = 5.847 (5) Å	Mo *K*α radiation, λ = 0.71073 Å
*b* = 8.981 (7) Å	Cell parameters from 1387 reflections
*c* = 11.630 (9) Å	θ = 2.2–22.6°
α = 80.456 (11)°	µ = 0.09 mm^−^^1^
β = 83.922 (11)°	*T* = 150 K
γ = 76.971 (12)°	Lath, colourless
*V* = 585.2 (8) Å^3^	0.60 × 0.20 × 0.10 mm

### Data collection

**Table:**

Bruker SMART 1000 CCD diffractometer	1965 independent reflections
Radiation source: sealed tube	1484 reflections with *I* > 2σ(*I*)
graphite	*R*_int_ = 0.029
Thin–slice ω scans	θ_max_ = 25.0°, θ_min_ = 2.4°
Absorption correction: multi-scan (*SADABS*; Sheldrick, 1996)	*h* = −6→6
*T*_min_ = 0.948, *T*_max_ = 0.991	*k* = −10→10
3801 measured reflections	*l* = −13→13

### Refinement

**Table:**

Refinement on *F*^2^	Secondary atom site location: difference Fourier map
Least-squares matrix: full	Hydrogen site location: difference Fourier map
*R*[*F*^2^ > 2σ(*F*^2^)] = 0.037	H atoms treated by a mixture of independent and constrained refinement
*wR*(*F*^2^) = 0.107	*w* = 1/[σ^2^(*F*_o_^2^) + (0.0421*P*)^2^ + 0.1501*P*] where *P* = (*F*_o_^2^ + 2*F*_c_^2^)/3
*S* = 1.09	(Δ/σ)_max_ < 0.001
1965 reflections	Δρ_max_ = 0.18 e Å^−^^3^
301 parameters	Δρ_min_ = −0.19 e Å^−^^3^
5 restraints	Extinction correction: *SHELXTL* (Sheldrick, 2008), Fc^*^=kFc[1+0.001xFc^2^λ^3^/sin(2θ)]^-1/4^
0 constraints	Extinction coefficient: 0.052 (8)
Primary atom site location: structure-invariant direct methods	

### Special details

**Table:**

Experimental. Data for this structure are only measured to 96% completeness. A data collection strategy which did not account for the lack of symmetry in the diffraction pattern, is the likely cause. This was not noticed with sufficient time to permit collection of further data before the crystal was lost.

### Fractional atomic coordinates and isotropic or equivalent isotropic displacement parameters (Å^2^)

**Table:**

	*x*	*y*	*z*	*U*_iso_*/*U*_eq_	
O1	0.4309 (6)	1.0147 (4)	0.5120 (3)	0.0483 (9)	
H1O	0.315 (7)	0.978 (6)	0.502 (5)	0.058*	
O2	1.0294 (6)	0.9201 (4)	0.4955 (3)	0.0557 (10)	
O3	1.0845 (5)	0.3954 (3)	0.5608 (3)	0.0402 (8)	
O4	1.3793 (5)	0.4681 (4)	0.6296 (3)	0.0455 (9)	
O5	0.8953 (6)	1.1318 (4)	−0.1687 (3)	0.0503 (9)	
O6	0.1406 (6)	0.3215 (4)	0.1436 (3)	0.0439 (9)	
N1	0.8675 (6)	0.7100 (4)	0.5365 (3)	0.0334 (9)	
H1N	0.764 (6)	0.669 (5)	0.518 (4)	0.040*	
N2	0.6236 (6)	0.7839 (4)	0.3491 (3)	0.0300 (9)	
C1	0.5571 (9)	1.0436 (5)	0.4030 (4)	0.0432 (13)	
H1A	0.6378	1.1290	0.4046	0.052*	
H1B	0.4448	1.0774	0.3411	0.052*	
C2	0.7387 (8)	0.9025 (5)	0.3729 (4)	0.0316 (10)	
H2	0.8383	0.9341	0.3017	0.038*	
C3	0.8937 (7)	0.8432 (5)	0.4743 (4)	0.0339 (11)	
C4	1.0023 (8)	0.6337 (5)	0.6364 (4)	0.0346 (11)	
H4	1.1029	0.7024	0.6538	0.042*	
C5	1.1574 (7)	0.4863 (5)	0.6042 (4)	0.0323 (11)	
C6	1.5397 (9)	0.3267 (6)	0.6013 (6)	0.0562 (15)	
H6A	1.6247	0.3485	0.5253	0.084*	
H6B	1.4494	0.2480	0.5981	0.084*	
H6C	1.6525	0.2887	0.6616	0.084*	
C7	0.8368 (9)	0.5979 (6)	0.7456 (4)	0.0460 (13)	
H7	0.7446	0.5242	0.7279	0.055*	
C8	0.6626 (11)	0.7440 (7)	0.7733 (5)	0.0648 (17)	
H8A	0.7476	0.8143	0.7980	0.097*	
H8B	0.5476	0.7164	0.8363	0.097*	
H8C	0.5806	0.7950	0.7034	0.097*	
C9	0.9806 (11)	0.5195 (7)	0.8486 (5)	0.0624 (16)	
H9A	0.8751	0.4898	0.9155	0.094*	
H9B	1.0683	0.5910	0.8695	0.094*	
H9C	1.0911	0.4271	0.8273	0.094*	
C10	0.6022 (7)	0.7716 (5)	0.2420 (4)	0.0276 (10)	
C11	0.6881 (7)	0.8712 (5)	0.1377 (4)	0.0291 (10)	
C12	0.5383 (8)	1.0007 (5)	0.0839 (4)	0.0348 (11)	
H12	0.3817	1.0296	0.1167	0.042*	
C13	0.6118 (9)	1.0880 (5)	−0.0156 (4)	0.0384 (12)	
H13	0.5081	1.1778	−0.0499	0.046*	
C14	0.8421 (8)	1.0435 (5)	−0.0666 (4)	0.0346 (11)	
C15	0.9931 (8)	0.9167 (5)	−0.0132 (4)	0.0356 (11)	
H15	1.1500	0.8877	−0.0456	0.043*	
C16	0.9164 (7)	0.8321 (5)	0.0871 (4)	0.0340 (11)	
H16	1.0220	0.7444	0.1230	0.041*	
C17	1.1079 (10)	1.0729 (7)	−0.2357 (5)	0.0585 (16)	
H17A	1.1145	0.9648	−0.2431	0.088*	
H17B	1.2444	1.0794	−0.1962	0.088*	
H17C	1.1097	1.1342	−0.3137	0.088*	
C18	0.4816 (7)	0.6505 (4)	0.2194 (4)	0.0252 (9)	
C19	0.3365 (8)	0.5832 (5)	0.3054 (4)	0.0329 (11)	
H19	0.3161	0.6142	0.3807	0.039*	
C20	0.2210 (8)	0.4728 (5)	0.2849 (4)	0.0337 (11)	
H20	0.1234	0.4287	0.3455	0.040*	
C21	0.2486 (8)	0.4269 (5)	0.1752 (4)	0.0310 (10)	
C22	0.3962 (7)	0.4910 (5)	0.0874 (4)	0.0332 (11)	
H22	0.4196	0.4583	0.0126	0.040*	
C23	0.5078 (7)	0.6023 (5)	0.1104 (4)	0.0312 (10)	
H23	0.6050	0.6469	0.0500	0.037*	
C24	−0.0323 (9)	0.2656 (6)	0.2253 (5)	0.0464 (13)	
H24A	−0.1542	0.3531	0.2465	0.070*	
H24B	0.0428	0.2083	0.2956	0.070*	
H24C	−0.1039	0.1971	0.1899	0.070*	

### Atomic displacement parameters (Å^2^)

**Table:**

	*U*^11^	*U*^22^	*U*^33^	*U*^12^	*U*^13^	*U*^23^
O1	0.040 (2)	0.058 (2)	0.057 (2)	−0.0183 (17)	0.0078 (18)	−0.0313 (18)
O2	0.055 (2)	0.071 (3)	0.056 (2)	−0.040 (2)	−0.0084 (18)	−0.0110 (19)
O3	0.041 (2)	0.046 (2)	0.039 (2)	−0.0155 (16)	−0.0067 (15)	−0.0091 (16)
O4	0.0304 (19)	0.050 (2)	0.061 (2)	−0.0060 (15)	−0.0111 (16)	−0.0209 (17)
O5	0.067 (2)	0.0368 (19)	0.040 (2)	−0.0102 (17)	0.0073 (18)	0.0055 (16)
O6	0.047 (2)	0.040 (2)	0.052 (2)	−0.0200 (16)	−0.0022 (17)	−0.0141 (17)
N1	0.032 (2)	0.042 (2)	0.031 (2)	−0.0128 (18)	−0.0042 (17)	−0.0113 (18)
N2	0.032 (2)	0.032 (2)	0.030 (2)	−0.0117 (16)	0.0000 (16)	−0.0091 (16)
C1	0.053 (3)	0.036 (3)	0.045 (3)	−0.012 (2)	−0.011 (3)	−0.012 (2)
C2	0.035 (3)	0.034 (2)	0.029 (2)	−0.015 (2)	0.008 (2)	−0.011 (2)
C3	0.031 (3)	0.044 (3)	0.030 (3)	−0.011 (2)	0.000 (2)	−0.013 (2)
C4	0.035 (3)	0.043 (3)	0.029 (3)	−0.007 (2)	−0.005 (2)	−0.015 (2)
C5	0.032 (3)	0.045 (3)	0.024 (2)	−0.017 (2)	−0.0011 (19)	−0.003 (2)
C6	0.031 (3)	0.053 (4)	0.088 (5)	−0.003 (2)	−0.007 (3)	−0.028 (3)
C7	0.043 (3)	0.058 (3)	0.036 (3)	−0.002 (2)	−0.008 (2)	−0.013 (3)
C8	0.061 (4)	0.078 (4)	0.048 (4)	0.014 (3)	−0.009 (3)	−0.024 (3)
C9	0.068 (4)	0.071 (4)	0.043 (3)	0.006 (3)	−0.008 (3)	−0.021 (3)
C10	0.023 (2)	0.031 (2)	0.028 (2)	−0.0033 (18)	0.0026 (18)	−0.0067 (19)
C11	0.025 (2)	0.032 (2)	0.031 (3)	−0.0073 (19)	−0.0026 (19)	−0.008 (2)
C12	0.030 (3)	0.036 (3)	0.035 (3)	−0.002 (2)	0.003 (2)	−0.004 (2)
C13	0.047 (3)	0.030 (3)	0.034 (3)	−0.003 (2)	−0.002 (2)	0.000 (2)
C14	0.041 (3)	0.030 (3)	0.032 (3)	−0.014 (2)	0.002 (2)	0.003 (2)
C15	0.032 (3)	0.039 (3)	0.034 (3)	−0.009 (2)	0.005 (2)	−0.003 (2)
C16	0.030 (3)	0.034 (3)	0.035 (3)	−0.006 (2)	0.002 (2)	0.001 (2)
C17	0.063 (4)	0.064 (4)	0.039 (3)	−0.015 (3)	0.012 (3)	0.008 (3)
C18	0.026 (2)	0.023 (2)	0.026 (2)	−0.0029 (17)	−0.0012 (18)	−0.0047 (17)
C19	0.036 (3)	0.038 (3)	0.025 (2)	−0.009 (2)	0.001 (2)	−0.007 (2)
C20	0.036 (3)	0.036 (3)	0.030 (3)	−0.013 (2)	0.004 (2)	−0.004 (2)
C21	0.032 (3)	0.024 (2)	0.036 (3)	−0.0035 (19)	−0.001 (2)	−0.009 (2)
C22	0.035 (3)	0.034 (3)	0.033 (3)	−0.003 (2)	−0.005 (2)	−0.014 (2)
C23	0.025 (2)	0.035 (2)	0.033 (3)	−0.0083 (19)	0.0030 (19)	−0.005 (2)
C24	0.044 (3)	0.039 (3)	0.057 (4)	−0.015 (2)	−0.007 (3)	0.002 (3)

### Geometric parameters (Å, °)

**Table:**

O1—H1O	0.844 (11)	C9—H9A	0.980
O1—C1	1.414 (6)	C9—H9B	0.980
O2—C3	1.230 (5)	C9—H9C	0.980
O3—C5	1.201 (5)	C10—C11	1.496 (6)
O4—C5	1.329 (5)	C10—C18	1.493 (6)
O4—C6	1.462 (6)	C11—C12	1.388 (6)
O5—C14	1.362 (5)	C11—C16	1.393 (6)
O5—C17	1.438 (6)	C12—H12	0.950
O6—C21	1.366 (5)	C12—C13	1.372 (6)
O6—C24	1.430 (6)	C13—H13	0.950
N1—H1N	0.843 (11)	C13—C14	1.411 (7)
N1—C3	1.322 (6)	C14—C15	1.375 (6)
N1—C4	1.458 (6)	C15—H15	0.950
N2—C2	1.458 (5)	C15—C16	1.374 (6)
N2—C10	1.290 (5)	C16—H16	0.950
C1—H1A	0.990	C17—H17A	0.980
C1—H1B	0.990	C17—H17B	0.980
C1—C2	1.523 (6)	C17—H17C	0.980
C2—H2	1.00	C18—C19	1.392 (6)
C2—C3	1.515 (6)	C18—C23	1.389 (6)
C4—H4	1.00	C19—H19	0.950
C4—C5	1.505 (6)	C19—C20	1.383 (6)
C4—C7	1.548 (7)	C20—H20	0.950
C6—H6A	0.980	C20—C21	1.388 (6)
C6—H6B	0.980	C21—C22	1.400 (6)
C6—H6C	0.980	C22—H22	0.950
C7—H7	1.00	C22—C23	1.384 (6)
C7—C8	1.527 (7)	C23—H23	0.950
C7—C9	1.518 (7)	C24—H24A	0.980
C8—H8A	0.980	C24—H24B	0.980
C8—H8B	0.980	C24—H24C	0.980
C8—H8C	0.980		
			
H1O—O1—C1	109 (4)	H9A—C9—H9C	109.5
C5—O4—C6	116.0 (4)	H9B—C9—H9C	109.5
C14—O5—C17	117.3 (4)	N2—C10—C11	124.7 (4)
C21—O6—C24	117.7 (4)	N2—C10—C18	118.2 (4)
H1N—N1—C3	117 (3)	C11—C10—C18	117.1 (4)
H1N—N1—C4	119 (3)	C10—C11—C12	120.9 (4)
C3—N1—C4	124.0 (4)	C10—C11—C16	121.2 (4)
C2—N2—C10	119.0 (4)	C12—C11—C16	117.8 (4)
O1—C1—H1A	109.0	C11—C12—H12	119.3
O1—C1—H1B	109.0	C11—C12—C13	121.4 (4)
O1—C1—C2	112.7 (4)	H12—C12—C13	119.3
H1A—C1—H1B	107.8	C12—C13—H13	120.2
H1A—C1—C2	109.0	C12—C13—C14	119.7 (4)
H1B—C1—C2	109.0	H13—C13—C14	120.2
N2—C2—C1	110.7 (4)	O5—C14—C13	115.6 (4)
N2—C2—H2	108.9	O5—C14—C15	124.9 (4)
N2—C2—C3	111.3 (4)	C13—C14—C15	119.4 (4)
C1—C2—H2	108.9	C14—C15—H15	120.1
C1—C2—C3	108.2 (4)	C14—C15—C16	119.8 (4)
H2—C2—C3	108.9	H15—C15—C16	120.1
O2—C3—N1	124.3 (4)	C11—C16—C15	121.9 (4)
O2—C3—C2	119.7 (4)	C11—C16—H16	119.0
N1—C3—C2	116.0 (4)	C15—C16—H16	119.0
N1—C4—H4	109.2	O5—C17—H17A	109.5
N1—C4—C5	108.0 (3)	O5—C17—H17B	109.5
N1—C4—C7	110.9 (4)	O5—C17—H17C	109.5
H4—C4—C5	109.2	H17A—C17—H17B	109.5
H4—C4—C7	109.2	H17A—C17—H17C	109.5
C5—C4—C7	110.2 (4)	H17B—C17—H17C	109.5
O3—C5—O4	124.4 (4)	C10—C18—C19	121.7 (4)
O3—C5—C4	122.6 (4)	C10—C18—C23	120.9 (4)
O4—C5—C4	113.1 (4)	C19—C18—C23	117.4 (4)
O4—C6—H6A	109.5	C18—C19—H19	118.9
O4—C6—H6B	109.5	C18—C19—C20	122.2 (4)
O4—C6—H6C	109.5	H19—C19—C20	118.9
H6A—C6—H6B	109.5	C19—C20—H20	120.3
H6A—C6—H6C	109.5	C19—C20—C21	119.5 (4)
H6B—C6—H6C	109.5	H20—C20—C21	120.3
C4—C7—H7	107.9	O6—C21—C20	125.0 (4)
C4—C7—C8	111.1 (5)	O6—C21—C22	115.4 (4)
C4—C7—C9	110.0 (4)	C20—C21—C22	119.6 (4)
H7—C7—C8	107.9	C21—C22—H22	120.2
H7—C7—C9	107.9	C21—C22—C23	119.5 (4)
C8—C7—C9	111.8 (4)	H22—C22—C23	120.2
C7—C8—H8A	109.5	C18—C23—C22	121.8 (4)
C7—C8—H8B	109.5	C18—C23—H23	119.1
C7—C8—H8C	109.5	C22—C23—H23	119.1
H8A—C8—H8B	109.5	O6—C24—H24A	109.5
H8A—C8—H8C	109.5	O6—C24—H24B	109.5
H8B—C8—H8C	109.5	O6—C24—H24C	109.5
C7—C9—H9A	109.5	H24A—C24—H24B	109.5
C7—C9—H9B	109.5	H24A—C24—H24C	109.5
C7—C9—H9C	109.5	H24B—C24—H24C	109.5
H9A—C9—H9B	109.5		
			
C10—N2—C2—C1	98.9 (5)	C10—C11—C12—C13	−176.3 (4)
C10—N2—C2—C3	−140.8 (4)	C16—C11—C12—C13	−0.2 (6)
O1—C1—C2—N2	69.0 (5)	C11—C12—C13—C14	1.8 (7)
O1—C1—C2—C3	−53.1 (5)	C17—O5—C14—C13	−167.4 (4)
C4—N1—C3—O2	−2.2 (7)	C17—O5—C14—C15	11.6 (7)
C4—N1—C3—C2	179.3 (4)	C12—C13—C14—O5	176.5 (4)
N2—C2—C3—O2	169.6 (4)	C12—C13—C14—C15	−2.6 (7)
N2—C2—C3—N1	−11.8 (5)	O5—C14—C15—C16	−177.1 (4)
C1—C2—C3—O2	−68.6 (5)	C13—C14—C15—C16	1.9 (7)
C1—C2—C3—N1	110.0 (4)	C14—C15—C16—C11	−0.4 (7)
C3—N1—C4—C5	−113.3 (5)	C10—C11—C16—C15	175.6 (4)
C3—N1—C4—C7	125.8 (5)	C12—C11—C16—C15	−0.5 (6)
C6—O4—C5—O3	0.0 (7)	N2—C10—C18—C19	18.7 (6)
C6—O4—C5—C4	179.6 (4)	N2—C10—C18—C23	−162.3 (4)
N1—C4—C5—O3	−48.6 (6)	C11—C10—C18—C19	−160.8 (4)
N1—C4—C5—O4	131.9 (4)	C11—C10—C18—C23	18.2 (6)
C7—C4—C5—O3	72.8 (5)	C10—C18—C19—C20	179.0 (4)
C7—C4—C5—O4	−106.8 (4)	C23—C18—C19—C20	−0.1 (6)
N1—C4—C7—C8	−55.3 (5)	C18—C19—C20—C21	−0.2 (7)
N1—C4—C7—C9	−179.7 (4)	C24—O6—C21—C20	6.8 (6)
C5—C4—C7—C8	−174.9 (4)	C24—O6—C21—C22	−173.0 (4)
C5—C4—C7—C9	60.8 (5)	C19—C20—C21—O6	−178.7 (4)
C2—N2—C10—C11	0.1 (6)	C19—C20—C21—C22	1.1 (6)
C2—N2—C10—C18	−179.4 (4)	O6—C21—C22—C23	178.1 (4)
N2—C10—C11—C12	−95.5 (5)	C20—C21—C22—C23	−1.7 (6)
N2—C10—C11—C16	88.5 (6)	C21—C22—C23—C18	1.4 (6)
C18—C10—C11—C12	84.0 (5)	C10—C18—C23—C22	−179.5 (4)
C18—C10—C11—C16	−92.0 (5)	C19—C18—C23—C22	−0.4 (6)

### Hydrogen-bond geometry (Å, °)

**Table:**

Cg1 is the centroid of the C18–C23 ring.

**Table:**

*D*—H···*A*	*D*—H	H···*A*	*D*···*A*	*D*—H···*A*
O1—H1O···O2^i^	0.84 (1)	1.87 (2)	2.705 (5)	170 (6)
N1—H1N···N2	0.84 (1)	2.21 (4)	2.641 (5)	112 (4)
C6—H6B···O1^ii^	0.98	2.49	3.353 (6)	146.
C17—H17C···O3^iii^	0.98	2.53	3.410 (6)	149
C20—H20···O3^i^	0.95	2.46	3.222 (6)	137
C16—H16···Cg1^iv^	0.95	2.52	3.460 (6)	169

Symmetry codes: (i) *x*−1, *y*, *z*; (ii) *x*+1, *y*−1, *z*; (iii) *x*, *y*+1, *z*−1; (iv) *x*+1, *y*, *z*.
